# A Retrospective Review of the Natural Progression of Cardiac Vegetation

**DOI:** 10.7759/cureus.21606

**Published:** 2022-01-25

**Authors:** Mehakmeet Bhatia, Saleha Asghar, Roomana Khan, Vivek Kak

**Affiliations:** 1 Internal Medicine, Henry Ford Health System, Jackson, USA; 2 Internal Medicine, Henry Ford Allegiance Health, Jackson, USA; 3 Infectious Disease, Henry Ford Allegiance Health, Jackson, USA

**Keywords:** emboli, iv drug use, staphylococcus aureus endocarditis, vegetation size, infective endocarditis

## Abstract

Introduction: Infective endocarditis (IE) is a life-threatening condition with an annual mortality of up to 40%. Vegetations are the hallmark of IE, however, factors that affect the initial size and changes in size remain unclear. Our study aims to investigate the natural history of cardiac vegetation, including changes in size and/or resolution with adequate treatment, and to analyze factors that influence size and potential for persistence.

Material and methods: We conducted a retrospective review of 102 patients admitted with native-valve endocarditis at Henry Ford Health System from September 1, 2017, to June 30, 2019. We included patients treated with six weeks of intravenous antibiotics who had both a diagnostic and a follow-up echocardiogram after antibiotic completion.

The primary outcome was the change in vegetation size. Secondary measures included pathogen identification, valve involvement, number of complications, associated IV drug use, and co-infection with hepatitis B/C.

Results: Of the 102 patients reviewed, 30 patients matched the inclusion criteria. There was a significant decrease in vegetation size after adequate antibiotic treatment. However, complete resolution was not often seen. A statistically significant relationship was seen between vegetation size, IV drug use, and Staphylococcal species (including both methicillin-susceptible *Staphylococcus aureus* [MSSA] and methicillin-resistant *S. aureus* [MRSA]), whereas a history of hepatitis B or C was not significantly related to vegetation size.

Conclusion: Large vegetation may predict a higher risk of embolic complications and can be reduced with IV antibiotics, although complete resolution is not likely. IV drug use and Staphylococcal endocarditis influence vegetation size and embolic complications. We argue that these subgroups should be prioritized for early surgical intervention.

## Introduction

Infective endocarditis (IE) is a complex endovascular infection secondary to endothelial damage with potentially life-threatening complications [1,2]. Areas of valvular coaptation, which are rough central areas on the surface of a valve, are most commonly affected, including the ventricular sides of aortic and pulmonic valves, and the atrial sides of mitral and tricuspid valves (Keifer). Endocardial damage attracts platelets and fibrin deposition to the site of injury, where the bacteria colonize, leading to the formation of infected vegetations [2-4]. The presence of vegetation, therefore, constitutes a major criterion as part of Duke’s criteria in the diagnosis of infective endocarditis. It is important to note that vegetation size has been seen as a predictor of embolic complications and mortality [5-8]. In fact, current AHA guidelines emphasize vegetation length greater than 10 mm as a cut-off for surgical intervention and recommend a follow-up transthoracic echocardiogram (TTE) after antimicrobial therapy is completed. Given that embolic complications contribute to a large mortality burden, factors such as vegetation size that largely affect embolic processes need to be studied in more detail.

The factors that impact the initial vegetation size in patients with native-valve infective endocarditis (NVIE) are poorly understood. There is a debate regarding the prognostic implications of vegetation length at the initiation and completion of treatment. The published literature suggests that vegetation size tends to reduce with antimicrobial therapy for at least six weeks; however, there is usually persistent residual vegetation in most cases [6,9]. The literature is lacking, though, in describing the factors that may predict increased growth and persistence of vegetations. In addition, the impact of hepatitis B or C on the vegetation size has not been explored, although a few case reports and one analysis show the co-existence of hepatitis in patients with tricuspid valve endocarditis and IV drug use [10-12].

We performed a retrospective analysis of patients with native valve endocarditis to assess the natural history of cardiac vegetation with adequate antimicrobial therapy. The primary objective of this study is to describe the factors that influence vegetation size and persistence, including but not limited to pathogen species, valve involvement, and to assess secondary complications (embolic or other).

## Materials and methods

We used a G*computational analysis to determine a sample size of 150 to provide adequate descriptive information for our primary and secondary outcomes. In addition, given the limited resources and time, we limited our search to a time period of just under two years. We performed a retrospective review of 102 patients discharged from the Henry Ford Health System with a diagnosis of NVIE from September 1, 2017, to June 31, 2019. We used the electronic health record to include patients 18 years or older admitted for native-valve endocarditis as defined by Duke’s criteria. Patients were included if they completed at least six weeks of IV antibiotics and had both an initial and follow-up echocardiogram completed.

Individuals younger than 18 years of age, those with prosthetic-valve endocarditis, pregnant patients, prisoners, and individuals with mortality <30 days from diagnosis were all excluded, mainly due to limited sample size as well as poor follow-up for echocardiograms, particularly in the inmate sub-group. The majority of our patients underwent TTE rather than transesophageal echocardiogram (TEE); stratified analysis was not completed because the sample size for TEE would be very small. Echocardiographic data were obtained by two certified readers. The primary outcome was the change in vegetation size, measured as cross-sectional area (height × width) on the follow-up echocardiogram. Secondary outcomes included the specific pathogen identified, valve involvement, the number and type of complications, associated intravenous drug use, and any co-infection with hepatitis B or C.

Continuous variables were expressed as mean. Nominal values were expressed as numbers. Unless otherwise noted, the test used for significance was an independent sample t-test. A lone Welch T-test was used to analyze the change in the size of vegetation on the echocardiogram. A value of p < 0.05 was considered significant.

## Results

We reviewed the charts of 102 patients admitted to Henry Ford Health System with a diagnosis of infective endocarditis. Out of the cases reviewed, 31 patients fulfilled all of the inclusion criteria, including having both initial and follow-up echocardiographic testing. Among these 31 patients, 18 were female and 13 were males. The mean age was 51.16 years.

Primary outcome

We noted an initial mean vegetation area of 1.70 cm^2^ (SD 2.12). The follow-up size after six weeks was significantly lower at 0.78 cm2 (SD 1.46; Table 1). The most common sites of vegetation were the tricuspid valve (n=7, mean 4.05 cm^2^), followed by the mitral valve (n=7, mean 0.73 cm^2^), aortic valve (n=5, mean 0.57 cm^2^), pulmonary valve (n=2, mean 0.64 cm^2^), and multiple cardiac vegetations or other cardiac vegetations (n=4, mean 1.54 cm^2^). The sample size of around 20 patients was too small to delineate the statistical difference between left and right-sided endocarditis. There was a statistically significant reduction in vegetation size of about 0.92 cm^2^ at the completion of antimicrobial treatment (p-value = 0.005; Table 1). Fourteen of the total cases had not been resolved by the end of treatment. Of note, the majority of patients did not undergo surgery given that they were not in overt valvular regurgitation or cardiac failure.

**Table 1:** Mean vegetation size before and after completion of antimicrobial therapy *p-value < 0.05

Size of largest vegetation (n=31)	Before treatment (cm^2^)	After treatment (cm^2^)	Mean change in growth (cm^2^)	[95% CI] (p-value)
Mean (cm^2^)	1.70	0.78	0.92	[0.30-1.53] (0.005)*
SD	2.12	1.46		

Secondary outcomes

An independent sample T-test was used for subgroup analysis. The initial size of vegetation was significantly larger in injection drug users (n=11, mean 3.11 cm^2^) when compared to non-IV drug users (n=16, mean 0.92 cm^2^; p-value = 0.026; Table 2). Patients who had embolic phenomena had significantly larger initial vegetations, 3.09 cm^2^, than those with no embolic complications, 0.83 cm^2^ (p-value = 0.013).

**Table 2:** Mean size of vegetation stratified by IV drug use, valvular and embolic complications *p-value < 0.05

Category (n)	Initial vegetation size (cm^2^)		Mean difference between two groups (cm^2^)	[95% CI] (p-value)
	Mean	SD		
IV drug use (11)	3.11	2.71	2.19	(0.026)*
No IV drug use (16)	0.92	1.21		
Valvular complications (17)	1.92	2.28	0.75	(0.379)
No valvular complication (10)	1.17	1.68		
Embolic complications (12)	3.09	2.59	2.26	(0.013)*
No embolic complications (16)	0.83	1.14		
Staph aureus group (12)	2.64	2.37	2.25	(0.001)*
Non-staph aureus group (15)	0.39	0.37		

We grouped patients with both methicillin-resistant *Staphylococcus aureus* (MRSA) and methicillin-susceptible *S. aureus* (MSSA) into one category, consisting of 12 cases, and all other pathogen species, including *Enterococcus faecalis* among others, into the non-*Staphylococcus aureus* category, comprising 15 cases. Patients with IE due to *S. aureus* had larger initial vegetations compared to those affected by non-Staphylococcal species (2.64 vs 0.39 cm^2^, respectively, p-value = 0.001). Patients with embolic complications also had larger vegetations compared to those without any emboli (3.09 vs 0.83 cm^2^, respectively, p-value 0.013). On the contrary, initial vegetation size did not differ significantly between patients with valvular complications versus those without (1.92 vs 1.17 cm^2^, respectively, p-value = 0.379).

We further stratified follow-up sizes after treatment based on IV drug use, pathogen species, and history of hepatitis. The *S. aureus* group showed a significantly larger reduction in vegetation size with treatment compared to the non-Staphylococcus group (mean difference of 0.94 cm^2^, p-value = 0.007; Table 3). On the other hand, vegetation sizes after treatment did not differ statistically based on the history of IV drug use or hepatitis B/C. We also noted that patients with Staphylococcal endocarditis had a significantly larger percent of embolic complications compared to those with non-Staphylococcal endocarditis (75% vs 18% of cases, p-value = 0.006).

**Table 3:** Vegetation size before and after treatment with antimicrobial therapy *p-value < 0.05

Variables (n=31) (n before treatment, n after treatment)	Size of initial vegetation size before treatment (cm^2^)		Vegetation size after treatment (cm^2^)		Mean difference between groups after treatment (cm^2^)	p-value
	Mean	SD	Mean	SD		
IV drug user (11, 12)	3.11	2.71	1.53	2.06	0.94	0.139
No IV drug use (16, 8)	0.92	1.21	0.58	1.38		
Staph aureus group (12, 9)	2.64	2.37	1.53	1.97	1.45	0.007*
Non-Staph aureus group (15, 11)	0.39	0.37	0.07	0.47		
History of Hepatitis B/C (4, 2)	3.27	3.29	1.23	2.27	0.36	0.660
No history of Hepatitis B/C (23, 18)	1.39	1.76	0.86	1.59		

## Discussion

Prognosis with initial versus residual vegetation

Our results show a statistically significant decrease in vegetation size after completing six weeks of IV antibiotics, although the vegetation did not completely resolve. Prior literature has shown similar results, with up to 70% of cases showing residual vegetation despite completion of antibiotic therapy [5,7-9]. According to published studies, residual vegetations did not increase the risk of embolism, death, or recurrence, except when they were > 10 mm in length or had increased in size after treatment [5]. Large initial vegetations (>1 cm or >1 cm^2^) have also been associated with greater embolic complications [7,13,14]. Therefore, we suspect that the initial vegetation size rather than residual vegetation is of more significance in predicting the risk of complications. We would rather argue that antimicrobial therapy should not be extended beyond six weeks if the size of vegetation has reduced, as persistent vegetation does not predict the risk of complications [5]. Moreover, large initial vegetation should motivate a more aggressive treatment approach at the outset, especially since the reduction in size (of at least 40%) has previously shown a decreased risk of mortality and embolic phenomenon [6].

Risk factors for large vegetations

The presence of vegetations, particularly those >1 cm or 1 cm^2^, is a prime factor leading to embolism, valvular regurgitation, and cardiac failure [6-8]. The relation between vegetation size and the embolic phenomenon is reinforced by our data. In addition, we argue that host-related factors, including the history of IV drug use and Staphylococcal endocarditis, predispose to large vegetations. In our analysis, patients with IV drug use and Staphylococcal​​​​​​ endocarditis had a mean vegetation size of 3.11 cm^2^ and 2.64 cm^2^, respectively, and patients with Staphylococcal​​​​​endocarditis had a significantly increased risk of embolic complications. In previous analyses, the history of IV drug use along with large vegetations has shown an increased risk of death from right-sided endocarditis as well as an embolic phenomenon [10,13,14].

Staphylococcal species have also been noted to independently predict the risk of the embolic phenomenon [8,14], though our data add to the literature in demonstrating that patients with Staphylococcal endocarditis also have larger vegetations, to begin with. Understanding the pathogenesis of vegetation formation helps delineate the effect of Staphylococcus species on vegetation size. Vegetation consists primarily of platelets and fibrin which deposit on the cardiac endothelium following some form of injury [3,4]. Staphylococcus and Streptococcus strains have been shown to enhance platelet aggregation more so than other pathogens, contributing to the faster growth of vegetations. In addition, bacterial density at the center of these vegetations is high and metabolically inert, which may decrease the antimicrobial effect, leading to the persistence of large vegetations [3]. It is likely that IV drug use and Staphylococcal go hand in hand in promoting vegetation growth, primarily due to the constant introduction of endovascular bacteria. Therefore, we would argue that it might be beneficial to prioritize patients with Staphylococcal endocarditis and/or those with a history of IV drug use for early surgical intervention and to follow their vegetation size more closely.

Implications for surgical intervention

Our results raise questions about the size criteria for surgical intervention in endocarditis. Traditionally, per AHA guidelines, early surgical intervention is recommended for vegetations larger than 10 mm [15]. In prior analysis [7], a size of 1 cm^2^ has been defined as large. However, this criterion is arbitrary at best. Our results show that larger vegetations (an average of 1.7 cm^2^ in our analysis, and at least 2 cm in a previous analysis) are associated with a greater percentage of embolic complications. Therefore, it is worth considering whether the cut-off for surgical intervention can be raised. Moreover, the risk of complications in endocarditis is likely multifactorial, with IV drug use and Staphylococcus species working in tandem to promote vegetation growth. Therefore, surgical intervention based on size alone in the absence of the above risk factors is likely not well supported. Further analysis with larger datasets is needed to elaborate on this concept.

Limitations

Our study has a few limitations, including, most importantly, a small sample size, which limits the certainty of our results. Due to the small sample size, we were unable to delineate differences in vegetation size based on left versus right-sided endocarditis or a history of hepatitis B or C. Our conclusions about the vegetation size in IV drug users and Staphylococcal endocarditis, though significant, are also of limited certainty. We also excluded patients with prosthetic valves, incarcerated and pregnant patients, further limiting the applicability of our results in these subgroups. Data on vegetation size were abstracted by a single reader, further limiting our precision due to a lack of inter-reader reliability.

## Conclusions

The current study reinforces that large vegetation size predicts greater embolic complications and that vegetation size shows significant reduction with at least six weeks of IV antimicrobial therapy. Our study brings to light IV drug use and *S. aureus* as potential predictors of large vegetations. We argue that patients with these risk factors should be followed closely and perhaps be prioritized for early surgical interventions. A history of hepatitis B or C, on the other hand, did not affect treatment outcomes in terms of vegetation size. Lastly, we propose that the current criteria of 10 mm for surgical intervention be re-visited using larger datasets as our dataset, though small, shows that a larger vegetation size is associated with embolic complications.
